# Examining karate and football perceptions and their links with athlete engagement and quality of life

**DOI:** 10.3389/fspor.2022.971677

**Published:** 2022-10-13

**Authors:** Teresa Limpo, Gabriela Rödel, Sid Tadrist

**Affiliations:** ^1^Faculty of Psychology and Education Sciences, University of Porto, Porto, Portugal; ^2^KWF Dynamic Karate, London, United Kingdom

**Keywords:** perceived benefits, aggressiveness-related risks, athlete engagement, quality of life, karate, football

## Abstract

The importance of perceptions as determinants of people's behavior has been well-established, but little is known about athletes' perceptions of their sport and the links of these perceptions with other correlates. In this study, we compared karate (*n* = 51) and football (*n* = 49) athletes' perceived benefits and aggressiveness risks from their sports and examined whether these perceptions predicted athletes' engagement and quality of life (QoL). Participants completed perception measures of karate and football, and engagement and QoL measures. Results showed that karateka perceived more benefits and fewer risks in karate than football, but footballers generally perceived equal benefits and risks in both sports. Both athlete groups perceived similar physical and psychological benefits in their own sport, but deemed physical benefits as prominent outcomes in the other sport. Notably, karateka's perceived benefits about karate predicted engagement directly and QoL indirectly *via* vigor. Overall, karate athletes' perceptions seemed to be relevant to experiencing fulfillment in training and general well-being.

## Introduction

The consequences of practicing sports are becoming well-known. Research showed several physical and psychological benefits of karate (1, 2) and football (3, 4). Also, evidence on sport aggression-related risks suggested that whereas martial arts may reduce aggressive behaviors (5), football may foster them (6). Knowing sport benefits and risks is important to inform current and future athletes. Still, according to the Theory of Planned Behavior (TPB) (7, 8), people's perceptions about the consequences of an activity are powerful predictors of intended and actual behaviors. Favorable perceptions about physical activity (including sports) seem positively associated with intentions and effective participation (9, 10).

Recognizing the lack of multidimensional instruments for assessing sport perceptions, Limpo and Tadrist (11) developed a scale to measure perceived physical, emotional, cognitive, and social benefits as well as aggression-related risks in karate and football. Besides providing validity evidence on this instrument, authors reported three main findings. First, physical benefits were perceived as salient outcomes of karate and football. Second, karate was perceived to have more psychological-related benefits and less aggressiveness risks than football. Third, perceptions varied according to involvement in physical activity. This study was however limited in two ways: it neither targeted athletes nor studied the predictive role of perceptions. Here, we aimed to overcome these gaps, by examining karateka and footballers' perceptions about karate and football as well as the contribution of these perceptions to athlete engagement and quality of life (QoL).

Initially defined in work settings (12), engagement in sport refers to a mindset of fulfillment during practice, characterized by strong mental resilience and high energy levels (vigor), full concentration and focus (absorption), and a sense of significance, enthusiasm, and pride (dedication). Some studies examining the antecedents of athlete engagement targeted basic psychological needs (13–15). To date, no study tested whether sport perceived benefits/risks contribute to athlete engagement. Yet, research from other fields and/or gauging related constructs alluded to this link. For example, perceived value in academic activities predicted undergraduates' school engagement (16); perceived monetary and non-monetary work benefits contributed to employees' work engagement (17), and perceived benefits of a digital library system predicted user absorption in library resources (18). In the sport setting, perceived value in marathon running among parent-child runners predicted their intentions to participate in this activity (19), and valuing sport practice was positively associated with correlates of engagement, such as involvement and resilience (20). Perceived risks in sport or other settings has been even less researched than perceived benefits. Some studies compared athletes' perceived injury risk across sports, including karate and football [e.g., Strotmeyer and Lystad (21)], but none has related it to engagement. We located one study showing that as non-athletes perceived levels of aggression in a sport increased, their willingness to be engaged in it declined (22).

Contrasting with sparse data relating athletes' perceptions and engagement, prior research has connected perceived sport-related benefits with aspects of QoL. Among exercisers at a fitness center, perceiving exercise to be beneficial or useful was associated with better exercise-related subjective experiences (23). The more college students perceived value in elite sports, the better their subjective well-being (24). Also, perceived social benefits of university-offered sports contributed to QoL (25). However, little is known about links between perceived risks in sports and QoL, even though research in work settings suggested that this association might be negative (26).

Besides perceptions, engagement may also be a likely predictor of QoL. A handful of studies showed that athlete engagement had, respectively, positive and negative associations with flow (13) and burnout (14, 15), which are deemed indicators of well-being in sport. To the best of our knowledge, there is no evidence on the link between athlete engagement and QoL. The picture is different in work settings, where a clear link between engagement and several aspects of QoL was found (27).

Collectively, available research supports the reasonable expectation that athletes may have different perceptions about sports, which may be linked to their engagement and QoL, also likely to be inter-related. Still, there is not compelling evidence corroborating these hypotheses, which were never tested in the same study with karate and football athletes.

This study had two major goals: (a) to examine whether perceived benefits and risks varied within and between sport (karate vs. football) and type of athlete (karateka vs. footballers); and (b) to test whether athletes' perceptions predicted engagement and QoL. Respectively, our hypotheses were as follows (a) based on Limpo and Tadrist (11), we expected karateka and footballers' perceptions about karate and football to be different, and (b) based on evidence from different settings showing perceptions-engagement (16, 20), perceptions-QoL (24, 25), and engagement-QoL links (27), we hypothesized that athletes perceiving more benefits and less risks in their sport would report more engagement, and, in turn, better QoL. By focusing on two types of athletes, we were able to ascertain whether this link was sport-general or sport-specific.

## Methods

### Participants

Participants were 100 athletes practicing karate or football in Portugal. Sample size was defined with a priori power analysis using G^*^Power 3 [Version 3.1.9.6; (28)], in which we specified: power = 80%, α = 0.05, analysis = repeated measures analysis of variance, and repeated-measures correlation = 0.40 [based on Limpo and Tadrist (11)]. A minimum of 96 participants was suggested to detect small effects ($\eta_{\text{p}}^{2}$ = 0.015), as reported by Limpo and Tadris (11). No power analysis was performed for the moderated mediation analysis as confidence intervals were built through bootstrapping (sample set to 5,000).

The karate group included 51 athletes (80% males) with a mean age of 38.55 years (*SD* = 13.81). The football group included 49 athletes (84% males) with a mean age of 21.88 years (*SD* = 7.48). Table 1 presents a characterization of both groups, which differed in terms of age, educational level, years of experience, and weekly training hours.

**Table 1:** Characterization and comparison of the karate and football groups.

	**Measures**	**Karate athletes (*n* = 51)**	**Football athletes (*n* = 49)**	**Comparison**
**Age**
	Mean (*SD*)	38.55 (13.81)	21.88 (7.48)	*t* = −7.55
	Range	18–65	17–49	*p* < 0.001
**Gender**
	Male (%)	41 (80%)	41 (84%)	x^2^ = 0.18
	Female (%)	10 (20%)	8 (16%)	*p* = 0.67
**Educational level**
	High school or below	26 (51%)	36 (61%)	x^2^ = 5.37
	Graduation or above	25 (49%)	13 (39%)	*p* = 0.02
**Karate graduation**
	4th kyu or below	6 (12%)	–	
	Between 3rd and 1st kyu	11 (21%)	–	
	1st dan	13 (25%)	–	
	2nd dan	10 (20%)	–	
	3rd dan	6 (12%)	–	
	4th dan or above	5 (10%)	–	
**Football involvement**
	Amateur	–	27 (55%)	
	Semi-professional	–	19 (39%)	
	Professional	–	2 (4%)	
	Ex-professional	–	1 (2%)	
**Instructor/coach functions**
	No	35 (67%)	39 (80%)	x^2^ = 1.56
	Yes	16 (31%)	10 (20%)	*p* = 0.21
**Years of practice**
	Mean (*SD*)	20.02 (12.68)	13.02 (7.94)	*t* = −3.32
	Range	1–47	1–40	*p* < 0.001
**Weekly training hours (last 6 months)**
	Mean (*SD*)	2.37 (1.64)	6.20 (2.37)	*t* = 5.19
	Range	0–6	0–24	*p* < 0.001
**Competition experience**
	None	22 (43%)	2 (4%)	*t* = 1.51
	Low (< 10 times)	7 (14%)	22 (45%)	*p* = 0.13
	Moderate (between 10 and 25 times)	5 (10%)	15 (31%)	
	High (25 times or more)	17 (33%)	10 (20%)	

### Measures

#### Perceptions about karate and football

We used the Portuguese Perceived Benefits and Aggressiveness Risks Scale (PBAR Scale), developed by Limpo and Tadrist (11). This is composed of five 3-item factors measuring perceived physical, emotional, social, and cognitive benefits along with perceived aggression-related risks in karate and football. Athletes were asked to indicate the degree to which they perceived each statement to represent outcomes of each sport, using a 5-point scale, from 1 (*totally disagree*) to 5 (*totally agree*). Cronbach's alphas for the karate/football versions were: 0.86/0.79 for physical benefits, 0.91/0.83 for emotional benefits, 0.74./0.80 for social benefits, 0.87/0.75 for cognitive benefits, and 0.76/0.79 for aggressiveness risks.

#### Athlete engagement

Engagement was measured with the 17-item Utrecht Work Engagement scale (29), validated to Portuguese by Simães and Gomes (30). For this study, the instrument was adapted to the sport context [for a similar procedure, see Martínez-Alvarado et al. (15) and Scotto di Luzio et al. (31)] by replacing the word “working” by “training” or “job” by “sport” (e.g., the original item “Time flies when I'm *working*” was changed to “Time flies when I'm *training*”). The instrument is composed of three engagement dimensions: vigor (6 items), absorption (6 items), and dedication (5 items). Athletes were asked to indicate how often they experienced the situations described, using a 5-point scale, from 1 (*almost never*) to 5 (*most of the time*). Cronbach's alphas were 0.80 for vigor, 81 for dedication, and 0.77 for absorption.

#### QoL

We used the EUROHIS-QOL-8 (32), validated to Portuguese by Pereira et al. (33). This is an 8-item unifactorial scale based on the WHOQOL-BREF (34), tapping psychological, physical, social, and environmental life domains. Athletes were asked to respond to each question using an individualized 5-point scale (e.g., ranging from “*not at all*” to “*completely*”). Cronbach's alpha was 0.80.

### Procedure

The study was implemented online with the LimeSurvey software and invitations to participate were spread *via* social media and sport clubs. After reading study goals, athletes were asked to provide a consent agreement, using a click-if-you-agree system. Those who agreed to participate were given access to the survey. The study was approved by the Ethics Committee of the first author's university.

## Data analysis plan

### Comparison of perceptions of football and karate

First, we examined the skewness and kurtosis of all variables separately by type of athlete. Respectively, values below |3| and |10| were considered as indicative of no severe deviations from the normal distribution (35). Afterwards, we conducted a 2 (Sport targeted [karate, football]) x 2 (Athlete [karateka, footballers]) x 5 (Perceptions [physical benefits, emotional benefits, social benefits, cognitive benefits, aggressiveness risks]) analyses of variance (ANOVA) with repeated measures in the first and last factors. When the sphericity assumption was violated, we used the Greenhouse-Geisser procedure. Considering an alpha level of 0.05, significant interactions were examined with tests of simple effects, followed-up through pairwise comparisons with Bonferroni correction. Preliminary 2 x 2 x 5 ANCOVAs introducing as covariates the sociodemographic characteristics or training-related features (hereafter referred as control variables), showed no main effects or interactions involving these variables. Thus, they were not introduced in the main ANOVA.

### Contribution of sport perceptions to engagement and QoL

As first preliminary step, separately for karate and football athletes, we inspected the correlations between all variables. Anticipating significant correlations involving control variables, these were accounted for in the subsequent analyses.

Second, stepwise regression analyses were used to examine the contribution of karate or football perceptions on athlete engagement and QoL, above and beyond control variables and type of athlete. For each dependent variable, we conducted two regression analyses with the same predictors on Step 1 (control variables plus type of athlete, which was dummy coded: 0 = footballers, 1 = karateka), but different predictors on Step 2. Whereas in one analysis we entered the main effects of karate perceptions and their interactions with type of athlete, in the other we added the main effects and interactions of football perceptions. To assure a participants/predictors ratio above 10 (36), we created a composite score labeled “overall benefits” by averaging athletes' perceived physical, emotional, social, and cognitive benefits in karate or football.

Finally, we used the PROCESS macro for SPSS version 3.5 (37) to test the presence of moderated mediation (38), that is, whether athlete engagement mediated the link between perceptions and QoL and whether this mediating effect was moderated by athlete type. Separate analyses were conducted to examine the contribution of perceived benefits/risks about karate/football, introducing age, education level, years of practice, and weekly training hours as covariates. The composite score combining all perceived benefits was used.

## Results

### Comparison of perceptions of football and karate

An inspection of skewness and kurtosis values showed no severe deviations from the normal distribution. Means and standard deviations by group are presented in Table 2. The analysis revealed main effects of sport *F*_(1,98)_ = 10.29, *p* = 0.002, $\eta_{\text{p}}^{2}$ = 0.10, and perceptions, *F*_(1.79,174.99)_ = 327.44, *p* < 0.001, $\eta_{\text{p}}^{2}$ = 0.77; two 2-way interactions between sport and athlete, *F*_(1,98)_ = 35.13, *p* < 0.001, $\eta_{\text{p}}^{2}$ = 0.26, and between sport and perceptions, *F*_(1.85,181.29)_ = 48.49, *p* < 0.001, $\eta_{\text{p}}^{2}$ = 0.33; and a 3-way interaction, *F*_(1.85,181.29)_ = 76.50, *p* < 0.001, $\eta_{\text{p}}^{2}$ = 0.44, described next.

**Table 2:** Means and standard deviations for perceptions, engagement, and quality of life for karate and football athletes.

**Measures**	**Karate athletes (*****n*** = **51)**		**Football athletes (*****n*** = **49)**	
	***M***	***SD***	***M***	***SD***
**Perceptions about karate**
Physical benefits	4.85	0.23	4.37	0.93
Emotional benefits	4.78	0.38	4.20	1.02
Social benefits	4.75	0.41	4.09	1.06
Cognitive benefits	4.66	0.48	3.86	1.02
Aggressiveness risks	1.34	0.63	2.27	0.96
**Perceptions about football**
Physical benefits	3.91	0.84	4.52	0.49
Emotional benefits	3.50	0.87	4.47	0.50
Social benefits	3.40	0.79	4.44	0.61
Cognitive benefits	3.50	0.83	4.10	0.70
Aggressiveness risks	2.76	1.10	2.24	0.88
**Athlete engagement**
Vigor	4.36	0.56	4.19	0.51
Absorption	4.43	0.59	4.52	0.42
Dedication	4.64	0.51	4.57	0.44
Quality of Life	4.17	0.43	4.02	0.45

#### Differences between sports

Karateka perceived karate as having statistically significant more physical, emotional, social, and cognitive benefits as well as less risk than football (*F*s > 51.77, *p*s < 0.001, $\eta_{\text{p}}^{2}$ > 0.34). Considering footballers, only one statistically significant difference was found: they perceived football to have more social benefits than karate (*F* = 5.82, *p* = 0.02, $\eta_{\text{p}}^{2}$ = 0.06).

#### Differences between athletes

In comparison to footballers, karateka perceived statistically significant more physical, emotional, social, and cognitive benefits (*F*s > 12.59, *p*s < 0.001, $\eta_{\text{p}}^{2}$ > 0.11) and less risks (*F* = 32.49, *p* = 0.001, $\eta_{\text{p}}^{2}$ = 0.25) in karate. The opposite pattern was found for football, in which football athletes perceived more physical, emotional, social, and cognitive benefits (*F*s > 15.44, *p*s < 0.001, $\eta_{\text{p}}^{2}$ > 0.13) and less risks (*F* = 6.77, *p* = 0.01, $\eta_{\text{p}}^{2}$ = 0.07) than karate athletes.

#### Differences between perceptions

There were statistically significant differences between perceptions across both sports and athlete types, *F*s < 19.54, *p*s < 0.001, $\eta_{\text{p}}^{2}$ > 0.45. Karateka perceived karate to have similar physical, emotional, social, and cognitive benefits (*t*s < 2.75, *p*s > 0.07), but perceived football as having more physical (ts > 4.29, *p*s < 0.001) than all other benefits, with no statistically significant differences between them (*t*s < 1.24, *p*s = 1.00). Footballers perceived football to have similar physical, emotional, and social benefits (*t*s < 0.91, *p*s = 1.00) and less cognitive benefit (*t*s > 3.54, *p*s > 0.001), but perceived karate to have statistically significant more physical than emotional and social benefits, which were deemed significantly higher than cognitive ones (ts > 3.21, *p*s < 0.02). Consistently across athletes and sports, emotional and social benefits was perceived to be similar, and all benefits were deemed higher than risks (*t*s > 9.14*, p*s < 0.001).

### Contribution of sport perceptions to engagement and QoL

Table 3 presents correlations between all variables, conducted as a preliminary step before the regression analyses. Karateka's age was linked to karate perceived benefits and engagement (0.30 < *r*s < 0.51), and their years of experience were negatively associated football perceived benefits (−0.35 < *r*s < −0.58). For footballers, there were correlations between age and vigor (*r* = −0.29), weekly training hours and QoL (*r* = 0.29), and some control variables and perceptions (−0.28 < *r*s < −0.34). In general, athletes' perceived benefits in their own sport were correlated with each other (0.34 < *r*s < 0.72) and with engagement (0.28 < *r*s < 0.70), which was related to QoL (0.34 < *r*s < 0.44).

**Table 3:** Bivariate correlations for karate athletes (above the diagonal) and football athletes (below the diagonal).

**Variables**	**1**	**2**	**3**	**4**	**5**	**6**	**7**	**8**	**9**	**10**	**11**	**12**	**13**	**14**	**15**	**16**	**17**	**18**	**19**	**20**
**Control Variables**
1. Age		**0.39**	**0.5**	0.14	**0.44**	**0.40**	**0.42**	**0.37**	**0.30**	0.06	−0.26	−0.24	−0.24	−0.27	−0.17	0.18	**0.35**	**0.46**	**0.51**	0.19
2. Educational level	**0.53**		0.18	−0.03	0.14	0.02	0.20	0.04	0.18	0.16	−0.03	0.05	−0.08	−0.02	−0.04	0.13	−0.13	−0.05	−0.06	0.04
3. Years of experience	**0.86**	**0.39**		**0.28**	0.16	0.19	0.13	0.05	0.19	0.20	**−0.54**	**−0.58**	**−0.51**	**−0.46**	**−0.35**	0.24	0.16	0.23	0.17	0.08
4. Weekly training hours	**−0.40**	−0.22	−0.26		0.09	0.20	0.06	−0.03	0.12	−0.11	−0.24	−0.16	−0.15	**−0.29**	−0.24	0.17	0.16	0.14	0.19	0.003
**Karate perceptions**
5. Benefits (overall)	0.09	0.13	−0.07	−0.26		**0.82**	**0.85**	**0.78**	**0.88**	< 0.001	0.03	0.01	−0.03	−0.03	0.19	0.08	**0.57**	**0.66**	**0.69**	0.22
6. Physical benefits	0.12	0.07	−0.02	−0.14	**0.94**		**0.64**	**0.61**	**0.62**	−0.01	−0.01	−0.03	−0.09	−0.15	0.21	0.12	**0.53**	**0.67**	**0.70**	0.15
7. Emotional benefits	0.05	0.08	−0.12	−0.20	**0.96**	**0.90**		**0.49**	**0.72**	0.01	0.01	0.01	0.004	−0.05	0.08	0.13	**0.38**	**0.48**	**0.57**	0.02
8. Social benefits	0.09	0.16	−0.05	**−0.34**	**0.95**	**0.85**	**0.89**		**0.51**	−0.14	−0.05	−0.12	−0.12	−0.02	0.09	−0.06	**0.62**	**0.64**	**0.67**	0.25
9. Cognitive benefits	0.09	0.18	−0.06	**−0.28**	**0.93**	**0.82**	**0.85**	**0.86**		0.12	0.12	0.11	0.05	0.05	0.20	0.08	**0.42**	**0.47**	**0.46**	0.27
10. Aggressiveness risks	−0.02	−0.02	0.21	< 0.001	0.12	0.19	0.10	0.09	0.09		0.15	0.14	0.05	0.11	0.25	0.19	−0.17	−0.18	−0.15	−0.14
**Football perceptions**
11. Benefits (overall)	−0.20	−0.16	−0.27	0.14	0.22	0.15	0.17	0.23	0.26	**−0.37**		**0.87**	**0.91**	**0.89**	**0.85**	**−0.36**	−0.21	−0.19	−0.20	0.13
12. Physical benefits	−0.15	**−0.30**	−0.08	0.27	0.05	0.13	0.04	−0.02	0.06	−0.13	**0.71**		**0.74**	**0.65**	**0.66**	−0.21	−0.26	−0.24	−0.22	0.18
13. Emotional benefits	−0.21	−0.17	**−0.32**	−0.003	0.25	0.18	**0.28**	0.26	0.23	−0.21	**0.68**	**0.34**		**0.82**	**0.65**	**−0.28**	−0.18	−0.19	−0.19	0.04
14. Social benefits	−0.19	−0.03	−0.24	0.08	0.06	−0.01	0.04	0.15	0.02	**−0.33**	**0.78**	**0.47**	**0.41**		**0.69**	**−0.57**	−0.23	−0.24	**−0.28**	0.08
15. Cognitive benefits	−0.06	−0.04	−0.17	0.09	0.26	0.15	0.15	0.26	**0.41**	**−0.39**	**0.77**	**0.37**	**0.36**	**0.42**		−0.24	−0.09	−0.01	−0.03	0.16
16. Aggressiveness risks	−0.05	−0.08	0.16	0.14	**−0.30**	−0.15	**−0.31**	**−0.36**	**−0.32**	**0.46**	**−0.48**	−0.11	**−0.52**	**−0.42**	**−0.36**		0.02	0.09	0.15	−0.23
**Athlete engagement**
17. Vigor	**−0.29**	−0.25	−0.16	0.06	−0.04	−0.09	−0.08	−0.05	0.08	−0.09	0.25	**0.28**	0.14	0.13	0.20	−0.27		**0.84**	**0.81**	**0.39**
18. Absorption	−0.23	−0.17	−0.15	−0.03	0.07	0.03	0.05	0.05	0.13	−0.07	**0.31**	**0.32**	**0.32**	0.15	0.18	−0.22	**0.76**		**0.87**	**0.34**
19. Dedication	−0.27	−0.09	**−0.30**	0.11	0.09	0.003	0.06	0.08	0.17	−0.24	**0.40**	0.23	**0.30**	**0.30**	**0.33**	**−0.44**	**0.76**	**0.68**		0.13
20. Quality of Life	−0.22	−0.12	−0.10	**0.29**	−0.09	−0.12	−0.06	−0.11	−0.06	−0.05	0.12	0.09	0.27	0.04	−0.002	−0.27	**0.41**	**0.28**	**0.44**	

Correlations equal to or above |0.28| are significant at an alpha level of 0.05 and are signaled in bold.

#### Predictive role of control variables and type of athlete

Step 1 of the regression analyses proved significant for vigor, absorption, and dedication, but not QoL (Table 4). Age (*b*s > 0.38) and educational level (*b*s > −0.21) predicted all engagement variables; years of experience predicted dedication (*b* = 0.25); and footballers reported more absorption than karateka (*b* = −0.30).

**Table 4:** Complete results of all regression models tested.

**Predictors**	**Vigor**				**Absorption**				**Dedication**				**Quality of Life**			
	***b***	***t***	***p***	**part corr**	***b***	***t***	***p***	**part corr**	***b***	***t***	***p***	**part corr**	***b***	***t***	***p***	**part corr**
**Step 1**	*R*^2^ = 0.12, *p* = 0.03				*R*^2^ = 0.14, *p* = 0.02				*R*^2^ = 0.17, *p* = 0.003				*R*^2^ = 0.08, *p* = 0.19			
Age	0.38	2.35	0.02	0.23	0.53	3.33	0.001	0.32	0.64	4.08	< 0.001	0.38	0.14	0.82	0.42	0.08
Educational level	−0.29	−2.65	0.01	−0.26	−0.26	−2.38	0.02	−0.23	−0.21	−2.01	0.05	−0.19	−0.05	−0.40	0.69	−0.04
Years of experience	−0.06	−0.49	0.63	−0.05	−0.08	−0.65	0.52	−0.06	−0.25	−2.01	0.05	−0.19	−0.04	−0.27	0.79	−0.03
Weely training hours	0.09	0.78	0.44	0.08	0.04	0.40	0.69	0.04	0.16	1.52	0.13	0.14	0.23	2.00	0.05	0.20
Athlete (0 = footballer, 1 = karateka)	0.06	0.45	0.65	0.04	−0.30	−2.33	0.02	−0.22	−0.11	−0.87	0.38	−0.08	0.22	1.68	0.10	0.17
**Step 2—Perceptions about karate**	Δ*R*^2^ = 0.16, *p* = 0.001				Δ*R*^2^ = 0.22, *p* < 0.001				Δ*R*^2^ = 0.21, *p* < 0.001				Δ*R*^2^ = 0.02, *p* = 0.66			
Age	0.09	0.53	0.60	0.05	0.18	1.17	0.25	0.10	0.30	1.99	0.05	0.17	0.03	0.15	0.89	0.02
Educational level	−0.26	−2.48	0.02	−0.22	−0.22	−2.24	0.03	−0.19	−0.19	−1.94	0.06	−0.16	−0.03	−0.23	0.82	−0.02
Years of experience	0.04	0.34	0.74	0.03	0.04	0.39	0.70	0.03	−0.12	−1.03	0.31	−0.09	0.01	0.10	0.92	0.01
Weekly training hours	0.05	0.43	0.67	0.04	0.01	0.07	0.95	0.01	0.14	1.45	0.15	0.12	0.21	1.75	0.08	0.18
Athlete (0 = footballer, 1 = karateka)	−0.19	−1.36	0.18	−0.12	−0.60	−4.56	< 0.001	−0.38	−0.41	−3.14	0.002	−0.26	0.11	0.70	0.49	0.07
Overall benefits	0.74	4.07	< 0.001	0.37	0.90	5.32	< 0.001	0.45	0.88	5.23	< 0.001	0.43	0.24	1.17	0.25	0.12
Aggressiveness risks	−0.15	−1.32	0.19	−0.12	−0.18	−1.66	0.10	−0.14	−0.19	−1.76	0.08	−0.15	−0.12	−0.92	0.36	−0.09
Athlete x Overall benefits	0.65	3.97	< 0.001	0.36	0.76	4.90	< 0.001	0.41	0.69	4.51	< 0.001	0.37	0.23	1.24	0.22	0.12
Athlete x Aggressiveness risks	−0.06	−0.56	0.58	−0.05	−0.09	−0.96	0.34	−0.08	0.02	0.23	0.82	0.02	−0.06	−0.52	0.61	−0.05
**Step 2—Perceptions about football**	Δ*R*^2^ = 0.05, *p* = 0.25				Δ*R*^2^ = 0.05, *p* = 0.24				Δ*R*^2^ = 0.08, *p* = 0.06				Δ*R*^2^ = 0.09, *p* = 0.06			
Age	0.38	2.36	0.02	0.23	0.53	3.32	0.001	0.32	0.64	4.15	< 0.001	0.38	0.13	0.82	0.42	0.08
Educational level	−0.26	−2.37	0.02	−0.23	−0.23	−2.08	0.04	−0.20	−0.18	−1.74	0.09	−0.16	−0.04	−0.38	0.71	−0.04
Years of experience	−0.10	−0.73	0.47	−0.07	−0.11	−0.77	0.44	−0.07	−0.30	−2.22	0.03	−0.20	0.07	0.52	0.61	0.05
Weekly training hours	0.07	0.65	0.52	0.06	0.01	0.07	0.94	0.01	0.13	1.22	0.23	0.11	0.28	2.45	0.02	0.24
Athlete (0 = footballer, 1 = karateka)	0.07	0.43	0.67	0.04	−0.29	−1.85	0.07	−0.18	−0.02	−0.12	0.90	−0.01	0.27	1.66	0.10	0.16
Overall benefits	0.03	0.19	0.85	0.02	0.17	1.02	0.31	0.10	0.11	0.72	0.48	0.07	0.07	0.42	0.67	0.04
Aggressiveess risks	−0.13	−1.16	0.25	−0.11	−0.02	−0.20	0.84	−0.02	−0.08	−0.74	0.46	−0.07	−0.29	−2.59	0.01	−0.25
Athlete x Overall benefits	−0.22	−1.83	0.07	−0.18	−0.27	−2.27	0.03	−0.22	−0.31	−2.71	0.01	−0.25	0.05	0.38	0.70	0.04
Athlete x Aggressiveess risks	−0.07	−0.54	0.59	−0.05	−0.13	−0.98	0.33	−0.09	0.01	0.06	0.95	0.01	−0.09	−0.67	0.51	−0.06

part corr, part correlation.

#### Predictive role of karate perceptions

When we added the main effects of karate perceptions and their interactions with type of athlete, there was a significant increase in the amount of variance explained in vigor, absorption, and dedication, but not in QoL (Table 4). The full model explained 28, 36, and 38% of the variance in vigor, absorption, and dedication, respectively. Significant predictors of vigor were educational level (*b* = −0.26), perceived benefits (*b* = 0.74), and the Athlete x Benefits interaction (*b* = 0.65). Significant predictors of absorption were educational level (*b* = −0.22), athlete type (*b* = −0.60), perceived benefits (*b* = 0.90), and the Athlete x Benefits interaction (*b* = 0.76). Significant predictors of dedication were age (*b* = 0.30), athlete type (*b* = −0.41), perceived benefits (*b* = 0.88), and the Athlete x Benefits interaction (*b* = 0.69). Athlete x Benefits interactions mean that greater perceived benefits about karate were associated with more vigor, absorption, and dedication only among karateka.

#### Predictive role of football perceptions

The inclusion of football perceptions and their interactions with type of athlete, led to no increase in the amount of variance explained in any outcome (Table 4).

#### Moderated mediation analyses

We found a single effect of moderated mediation involving vigor. For karateka (but not footballers) the perception of more benefits in karate was associated with better QoL through higher vigor, estimate = 0.48, bootstrap standard error = 0.16, 95% CI [0.18; 0.81]. No moderated mediation effects were found for football perceptions.

## Discussion

### Comparison of perceptions of football and karate

There were four main findings concerning karate and football athletes' perceptions. It is worth keeping in mind that, despite group differences in sociodemographic and training-related features, preliminary ANCOVAs showed that perceptions did not vary as a function of these variables.

First, between-group athlete comparisons showed that karateka (vs. footballers) perceived karate to bring them more benefits, whereas footballers (vs. karateka) perceived football to bring them more benefits. Though these findings may suggest endogroup favoritism (39), within-group comparisons advise caution in assuming that. Though karateka clearly perceived more benefits in karate than football, footballers only perceived football to have more social benefits than karate. Also, due to the few studies comparing actual karate and football benefits, it is difficult to infer whether athletes were overestimating the benefits of their own sport or not.

Second, karateka and footballers tended to perceive as much physical as psychological benefits in their own sport. Given available evidence showing that those are real benefits of karate and football (1–3), these athletes seem to have a richer knowledge about the benefits of their sport than other sports. This result extends prior findings with non-athletes, who perceived physical benefits as the most salient outcomes of karate and football, likely due to their limited knowledge about these sports (11). The same was observed here, when athletes judged the non-practiced sport. It seems that for people not practicing a specific sport, the physical dimension is deemed its core feature. Also, there was a tendency to devalue the cognitive benefits of karate and football, particularly evident among footballers. This finding matches those of Limpo and Tadrist (11), who noted that this devaluing contrasts with evidence-based cognitive gains of these sports (4, 40). Overall, there seems to be a need to raise people's awareness about the psychological (mainly cognitive) benefits of sports, in which they do not partake in.

Third, regardless of athlete type, social and emotional benefits were perceived to the same extent in both sports. Though this finding may question PBAR scale's discriminant validity, we believe it would be premature to conclude that. First, the connection between social and emotional aspects is well recognized (47), including in the study of perceived benefits of physical activity (41). Second, Limpo and Tadrist (11) showed clear differences between social and emotional benefits. Finally, in the present study, footballers did discriminate between these aspects, by deeming social (but not emotional) benefits in football to outweigh those in karate. Still, future studies may explore the comparative merits of studying perceived emotional and social benefits as separate or joint constructs.

Fourth, our results involving aggressiveness-related risks revealed that: (a) both sports were deemed to have larger benefits than risks; (b) karateka perceived more risks in football than karate, but footballers perceived equal risks between them; and (c) karateka perceived less risks in karate than footballers, while footballers perceived less risks in football than karateka. These findings partially align with Limpo and Tadrist (11), who found that perceived benefits of karate surpassed its risks, and that perceived karate risks were lower than perceived football risks. The different results mainly involve football and may be explained by the samples: athletes vs. non-athletes. Athletes may have underestimated the risks in their own sport, as it happened with Muay Thai fighters, concerning injury risks (21). Future research may examine the degree to which perceived risks in different sports match actual risks.

### Contribution of sport perceptions to engagement and QoL

After controlling for participants' age, education level, years of practice, and weekly training hours, we found that karateka's perceptions about karate predicted their engagement. The more benefits karateka perceived in their own sport, the more they reported high levels of vigor, absorption, and dedication. Specifically, they reported to have more energy and mental resilience while training, to be fully focused and happily engrossed in karate, and to feel strongly identified and enthusiastic about it. Karateka's perceptions about football did not predict their sport engagement, confirming the specificity of this perception-engagement link. Though past evidence hinted at this link (17), this study provides its first empirical demonstration. Determining the correlates of engagement is relevant because engagement is seen as a form of optimal functioning, associated with positive cognitive and emotional experiences (13, 14) and performance (42).

Although karate and football athletes' perceptions did not predict QoL directly, karateka's perceived benefits in karate did predict QoL indirectly through vigor. The more karateka perceived benefits in karate, the more energy they spent in this sport, and the better they felt about their lives. This is the first study linking core dimensions of athletes' lives in a mediating chain from perceptions to QoL *via* engagement, specifically, vigor. The prominent role of vigor in sport studies was also reported by Stolarski et al. (42), who found that this was the unique engagement dimension with a clear and positive relationship with running performance. The noticeable association of vigor with physical strength (43) may explain its central role in sport-related models and athlete samples, as those tested here and in the study conducted by Stolarskli et al. (42).

Two additional remarks are worth mentioning. First, karateka's perceived aggression risks in karate were not associated with either engagement or QoL. Likely, the perception of few risks in their sport diminished the relevance of this variable in relation to sport- and life-related outcomes. More research is needed to elucidate the potential role of perceived risks in sports, which may act as a more relevant predictor of intentions to become an athlete (22) rather than of engagement while being one. Second, the perception-engagement-QoL link was not observed among football players, indicating that this may be a sport-specific link. Its occurrence in karate but not in football may be related to the nature of these sports. Though both karate and football have a strong focus on physical skills (e.g., strength, speed), karate has an additional focus on the mind and the spirit (44). The degree to which these features relate to current findings is however open to inquiry. Future studies testing the perception-engagement-QoL in other sports seem warranted.

### Limitations and future research directions

Interpretations of these findings should consider four limitations. First, our data were obtained at a single time point and this study was correlational in nature. Thus, causality inferences should be avoided. More research is needed to replicate our results, through experimental and longitudinal tests of the mechanisms through which athletes' perceptions influence engagement and QoL. Second, we used a single-indicator approach, which did not model measurement error. Despite the validity and reliability of the instruments we used, it is advisable to cross-validate these findings with a multiple-indicator approach. Third, the groups of karate and football athletes differed in some characteristics. Though we statistically assured that our findings were not associated with these differences, future studies should aim for matched groups of athletes. Finally, because data was collected during the COVID-19 (January-February 2021), we cannot know whether results were influenced by the pandemic and will be replicated once it is over. However, the pattern of these findings aligned with pre-pandemic studies, and there is indication that the pandemic does not greatly threaten studies' external validity (45).

## Conclusion

A clear-cut message of this study is that athletes' perceptions matter in karate, but not in football. As shown here, karateka's perceived benefits about karate predicted vigor, which, in turn, predicted QoL. Still, footballers' perceptions played not predictive role either on engagement or QoL. These findings have a twofold implication. From a research viewpoint, a future avenue of inquiry shall aim not only to replicate these findings but also to gather evidence-based explanations. For example, which characteristics of karate and football are likely to explain the differential role of athlete's perceptions on engagement and QoL? From an applied viewpoint, sport psychologists should aim to gauge athletes' perceived benefits and risks about karate, and implement psycho-educational workshops highlighting its multiple benefits. By nurturing positive perceptions about karate, sport psychologists may be also boosting athletes' engagement and well-being. Given the pandemic's detrimental effects on athletes professional and personal lives (46), this seems particularly relevant in present times.

## Data availability statement

The raw data supporting the conclusions of this article will be made available by the authors, without undue reservation.

## Ethics statement

The studies involving human participants were reviewed and approved by Faculty of Psychology and Education Sciences of the University of Porto. The patients/participants provided their written informed consent to participate in this study.

## Author contributions

TL designed the study, oversaw the data collection and coding, analyzed and interpreted the data, and wrote the first version of the manuscript. GR helped in the preparation and implementation of the study. ST contributed to the design of the study and interpretation of the data. All authors reviewed the article and approved the submitted version.

## Conflict of interest

The authors declare that the research was conducted in the absence of any commercial or financial relationships that could be construed as a potential conflict of interest.

## Publisher's note

All claims expressed in this article are solely those of the authors and do not necessarily represent those of their affiliated organizations, or those of the publisher, the editors and the reviewers. Any product that may be evaluated in this article, or claim that may be made by its manufacturer, is not guaranteed or endorsed by the publisher.
